# Identification of Novel Glycolysis-Related Gene Signatures Associated With Prognosis of Patients With Clear Cell Renal Cell Carcinoma Based on TCGA

**DOI:** 10.3389/fgene.2020.589663

**Published:** 2020-12-18

**Authors:** Chengjiang Wu, Xiaojie Cai, Jie Yan, Anyu Deng, Yun Cao, Xueming Zhu

**Affiliations:** ^1^Department of Clinical Laboratory, The Second Affiliated Hospital of Soochow University, Suzhou, China; ^2^Department of Radiology, Affiliated Changshu Hospital of Soochow University, First People’s Hospital of Changshu City, Suzhou, China

**Keywords:** clear cell renal cell carcinoma, glycolysis-related gene, TCGA, prognosis, R programming language

## Abstract

**Objective:**

The purpose of the present study was to detect novel glycolysis-related gene signatures of prognostic values for patients with clear cell renal cell carcinoma (ccRCC).

**Methods:**

Glycolysis-related gene sets were acquired from the Molecular Signatures Database (V7.0). Gene Set Enrichment Analysis (GSEA) software (4.0.3) was applied to analyze glycolysis-related gene sets. The Perl programming language (5.32.0) was used to extract glycolysis-related genes and clinical information of patients with ccRCC. The receiver operating characteristic curve (ROC) and Kaplan–Meier curve were drawn by the R programming language (3.6.3).

**Results:**

The four glycolysis-related genes (B3GAT3, CENPA, AGL, and ALDH3A2) associated with prognosis were identified using Cox proportional regression analysis. A risk score staging system was established to predict the outcomes of patients with ccRCC. The patients with ccRCC were classified into the low-risk group and high-risk group.

**Conclusions:**

We have successfully constructed a risk staging model for ccRCC. The model has a better performance in predicting the prognosis of patients, which may have positive reference value for the treatment and curative effect evaluation of ccRCC.

## Introduction

Renal cell carcinoma (RCC) is one of the most common malignant tumors of the urinary system (Ferlay et al., 2010). Clear cell renal cell carcinoma (ccRCC) is the predominant RCC subtype, occupying 75% (Fan et al., 2015). Insidious onset of ccRCC and lack of specific symptoms and effective diagnostic and therapeutic tools result in an advanced stage and poor prognosis when diagnosed (Jonasch et al., 2014). ccRCC is not sensitive to radiotherapy and chemotherapy and exhibits an inherited predisposition to infiltrate and metastasize.(Miller et al., 2019). Molecular targeted therapy for advanced or metastatic ccRCC has notable curative effectiveness. However, the biological behavior of ccRCC is very complex and changeable (Ruan et al., 2019). At present, there is still a lack of effective molecular markers to evaluate the prognosis of patients with ccRCC and the effects of targeted drug therapy. The increasing drug resistance of metastatic ccRCC urgently needs to search biomarkers for diagnosis and prognosis (Makhov et al., 2018). Cancer cells mainly obtain energy through glycolysis to promote their growth. Inhibition of glycolysis has the effect of inhibiting proliferation and killing tumor cells. Glycolysis rate-limiting enzymes and hypoxia-inducible factors are expected to be new targets for tumor treatment (DeBerardinis et al., 2008; Dang et al., 2011). Therefore, it is of great significance to screen glycolysis-related genes associated with the prognosis of patients with ccRCC.

## Materials and Methods

### Clinical Information and mRNA Expression Dataset of Patients With ccRCC

The mRNA expression profiles were downloaded from the TCGA database^1^ (Wang et al., 2016). The clinical information of patients with ccRCC was also extracted from TCGA. The raw transcriptome expression data was collated by the Perl programming language (5.32.0) (Fourment and Gillings, 2008; Qiu et al., 2019). The transformation between mRNA name and identification (ID) was performed by R and Perl programming language. Besides, the R programming language (3.6.3) was used to extract and consolidate the clinical information, including survival time, survival state, age, gender grade, stage, and TNM stage.

### Gene Set Enrichment Analysis

Gene set enrichment analysis (GSEA) (4.0.3)^2^ was a tool for genome-wide expression profile data analysis, which detected gene sets rather than individual genes expression changes (Mootha et al., 2003). Therefore, GSEA could contain genes with small differences in expression and important functions, making the analysis more accurate and comprehensive (Subramanian et al., 2005). GSEA was used to determine whether *a priori* defined gene sets enriched in glycolysis-related functions and pathways showed significant differences between gene expression data of ccRCC and paracancerous samples. Glycolysis-related gene sets were downloaded from the Molecular Signatures Database (MSigDB V7.0)^3^ (Liberzon et al., 2011, 2015).

### Construction of Glycolysis-Related Gene Prognosis Model

The Perl programming language had also been applied to extract, filter, and combine the expression of glycolysis-related genes. A risk score staging model was established using the R programming language package survival function coxph (Zhang et al., 2019). R package “DEseq 2” was used to analyze the differentially expressed genes. Genes with a *p* < 0.05 were considered differentially expressed glycolysis-related genes. The differentially expressed glycolysis-related genes were combined with survival time and state to further analysis. Correlation analysis of gene expression data and survival data was carried out to find the glycolysis-related genes associated with prognosis. The genes with a *p* ≤ 10^–6^ were identified as glycolysis-related genes most significantly associated with prognosis. The risk score formula was described as follows: Risk score = expression of gene 1 × β1 + expression of gene 2 × β2 + … + expression of gene n × βn. According to the median value of patients’ risk score, patients with ccRCC were divided into high-risk and low-risk groups.

Semi-quantitative PCR was performed to detect the expression of four mRNAs. A total of 10 pairs of ccRCC and paracancerous tissue were collected between September and October 2020. Research samples were from Nanjing Drum Tower Hospital. All cases were diagnosed with the WHO histopathological classification of renal tumors in 2016. The experiment was approved by the Ethic Committee of the Second Affiliated Hospital of Soochow University. The primers for mRNAs were as follows. B3GAT3 forward primer GGTACGACTGTCCCAGACAC, reverse primer ATCTAACA GCAAGGGCAGGG; CENPA forward primer TGCGATGCTG TCTGGACTTT, reverse primer TGCTGCCACTAATGGTG AGG; AGL forward primer CCCCATTGCAGACTCTTGGA, reverse primer AACGCCAAAGTGCTCTGTCT; ALDH3A2 forward primer AGTCATTACTGTCCTTGGGGAA, reverse primer CTTCAGGAGCTCCGTGGTTT; ACTIN forward primer CTCCATCCTGGCCTCGCTGT, reverse primer GCTGT CACCTTCACCGTTCC. Total RNA was reverse transcribed into cDNA using PrimeScript^TM^ RT Master Mix (Takara Biotechnology Co., Ltd.) at 42°C for 30 min. qPCR was carried out using TB Green^TM^ Fast qPCR mix on a CFX96^TM^ Real-Time PCR Detection system (Bio-Rad Laboratories, Inc.). The mRNAs expressions were calculated by the 2^–ΔΔCq^ method. The thermocycling conditions were as follows: 95°C for 30 s, followed by 40 cycles of 95°C for 5 s and 60°C for 30 s.

### Statistical Analysis

The mRNA expression profiles were presented as raw data, and raw data were normalized by log2 transformation for further analysis. Differentially expressed genes were extracted with a *p* < 0.05. Univariate and multivariate regression analysis was performed on differentially expressed glycolysis-related genes to construct a risk staging model for ccRCC. “Survdiff” function was utilized to compare the survival differences between high-risk and low-risk groups. The mRNA expression data was presented as mean ± SD. *P* < 0.05 was considered to indicate a statistically significant difference. The software used for analysis was GraphPad Prism 7 (GraphPad Software, Inc.).

## Results

### Initial Screening of the Genes Using GSEA

**FIGURE 1:**
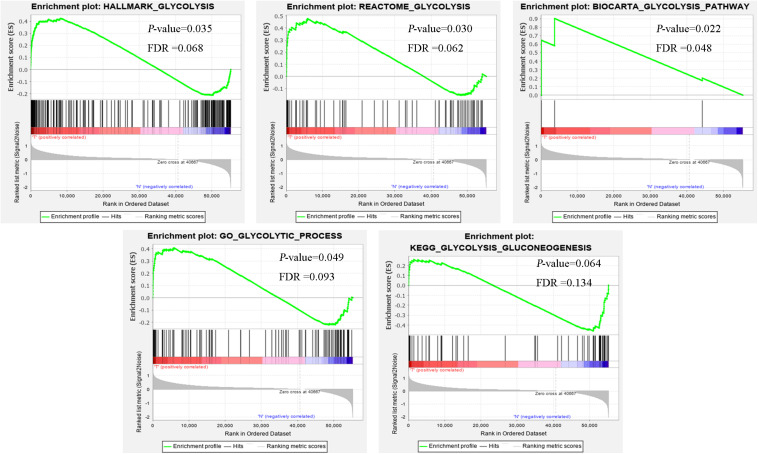
The GSEA analysis results of glycolysis-related gene sets (HALLMARK_GLYCOLYSIS, REACTOME_GLYCOLYSIS, BIOCARTA_GLYCOLYSIS_PATHWAY, GO_GLYCOLYTIC_PROCESS, and KEGG_GLYCOLYSIS_GLUCONEOGENESIS).

Clinical features of ccRCC patients were shown in Table 1. The mRNA expression profiles were from 72 paracancerous samples and 539 patients with ccRCC. Glycolysis-related gene sets mainly included “biocarta-glycolysis-pathway,” “go-glycolytic-process,” “hallmark-glycolysis,” “kegg-glycolysis-gluconeogenesis,” and “reactome-glycolysis.” GSEA was used for the transcriptome data to explore whether the glycolysis-related gene sets showed statistical differences between the ccRCC and adjacent normal tissues. Kegg-glycolysis-gluconeogenesis gene set had no significant difference between the ccRCC and adjacent normal tissues (*p* > 0.05). The other four gene sets had obvious differences in ccRCC and adjacent normal tissues (*p* < 0.05). GSEA results were shown in Figure 1. “biocarta-glycolysis-pathway,” “go-glycolytic-process,” “hallmark-glycolysis,” and “reactome-glycolysis” were selected for further analysis.

**TABLE 1:** Clinical features of ccRCC patients (*n* = 539) from TCGA database.

**Variables**	**Patients, *n* (%)**
**Sex**	
Male	346 (64)
Female	191 (36)
**Age, years**	
≤65	352 (66)
>65	185 (34)
**Stage**	
I	269 (50)
II	57 (11)
III	125 (23)
IV	83 (15.4)
unknown	3 (0.6)
**T stage**	
T1	275 (51)
T2	69 (13)
T3	182 (34)
T4	11 (2)
**N stage**	
N0	240 (45)
N1	17 (3)
N2	280 (52)
**M stage**	
M0	426 (79)
M1	79 (15)
MX	30 (5.6)
Unknown	2 (0.4)
**Grade**	
G1	14 (2.6)
G2	230 (42.9)
G3	207 (38.5)
G4	78 (14.5)
GX	5 (0.9)
Unknown	3 (0.6)

### Identification of Glycolysis-Related Genes Associated With the Prognosis of Patients With ccRCC

**FIGURE 2:**
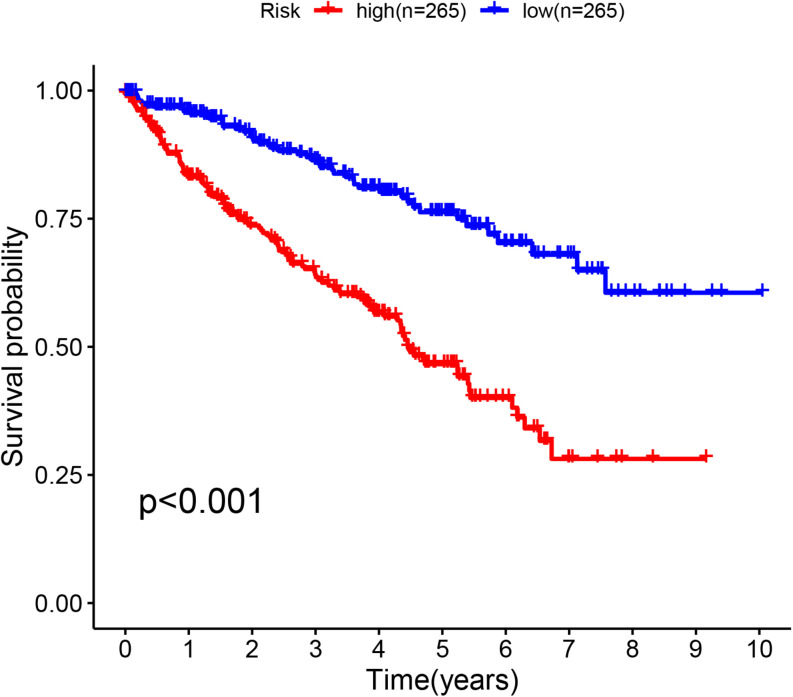
Kaplan–Meier curve for patients with ccRCC divided into the high-risk and low-risk groups.

**FIGURE 3:**
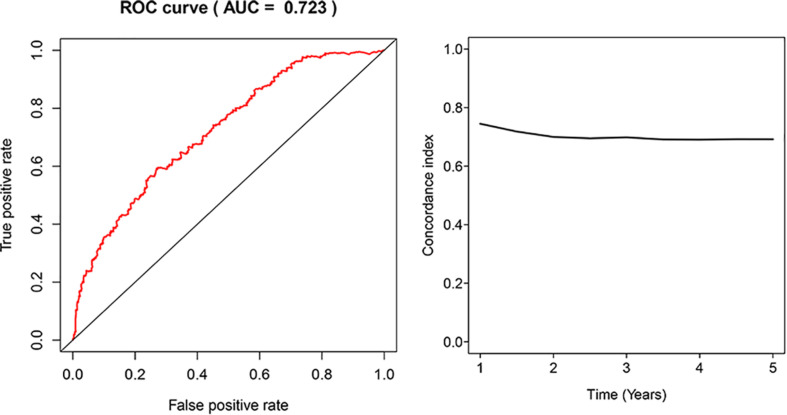
Receiver operating characteristic (ROC) analysis of the sensitivity and specificity of the risk score model and concordance index in a time-dependent Area Under the Curve (AUC) analysis.

**FIGURE 4:**
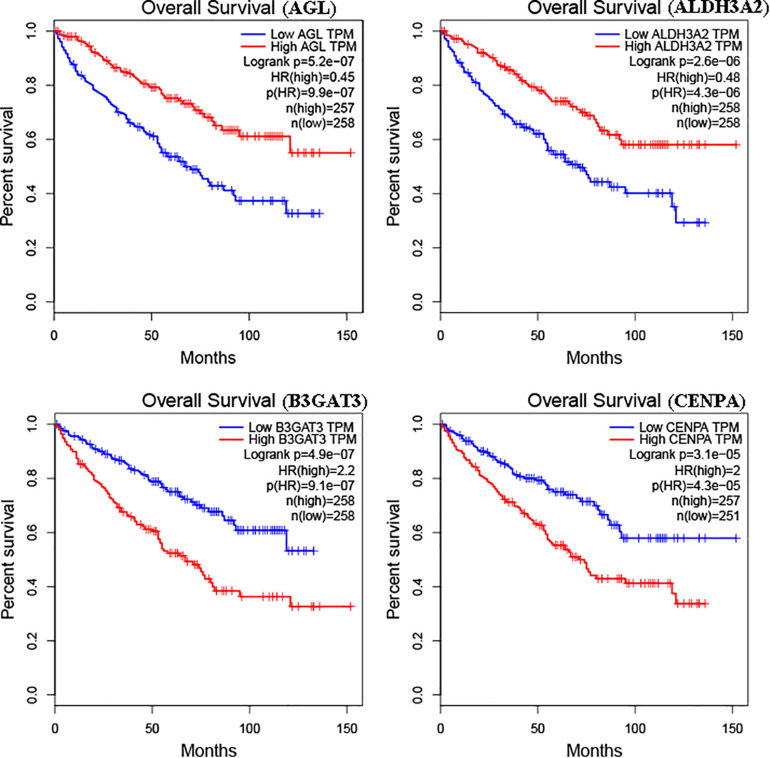
Kaplan–Meier curve for patients with ccRCC divided into high expression and low expression of mRNAs.

The glycolysis-related gene expressions were extracted from the glycolysis-related gene sets. Then, the 258 differentially expressed glycolysis-related genes with a *p* < 0.05 were acquired from paracancerous and ccRCC samples. Through the combination of glycolysis-related gene and survival time, we found 10 genes closely related to survival time and state of patients with ccRCC (*p* < 10^–6^). The 10 genes were processed by a stepwise multivariate Cox regression analysis. Finally, B3GAT3, CENPA, AGL, and ALDH3A2 were selected to establish a gene-based prognostic model (Table 2). Prognostic model were performed to evaluate the survival of each patients as follows: Risk score = 0.4097 × expression of B3GAT3 + 0.5956 × expression of CENPA + (−0.3792) × expression of AGL3 + (−0.1671) × expression of ALDH3A2. The patients with ccRCC were divided into high-risk and low-risk groups according to the risk score. The difference of survival probability between high-risk and low-risk groups was significant (*p* < 10^–3^, Figure 2). Evaluation of the accuracy of the glycolysis prognosis model was carried out by the receiver operating characteristic (ROC) curve. The 5 years area under the curve (AUC) of ROC (AUC = 0.732) showed that the model has an important diagnostic value in predicting the prognosis of patients with ccRCC (Figure 3). In addition, concordance index was all more than 0.7 to indicate that the model was of high reliability and high precision of forecasting (Figure 3). Through the GEPIA^4^, we verified that B3GAT3, CENPA, AGL, and ALDH3A2 were indeed closely related to the prognosis of patients with ccRCC (Figure 4).

**TABLE 2:** The detailed information of four prognostic mRNA significantly associated with survival in patients with ccRCC.

**Mrna**	**Ensemble ID**	**Location**	**Cox (β)**	**HR**
B3GAT3	ENSG00000149541	Chromosome 11: 62,615,296–62,622,154	0.4097	1.5064
CENPA	ENSG00000115163	Chromosome 2: 26,764,289–26,801,067	0.5956	1.8141
AGL	ENSG00000162688	Chromosome 1: 99,850,361–99,924,023	−0.3792	0.6844
ALDH3A2	ENSG00000072210	Chromosome 17: 19,648,136–19,685,760	−0.1671	0.8462

### Association Between Risk Scores and Outcomes of Patients With ccRCC

**FIGURE 5:**
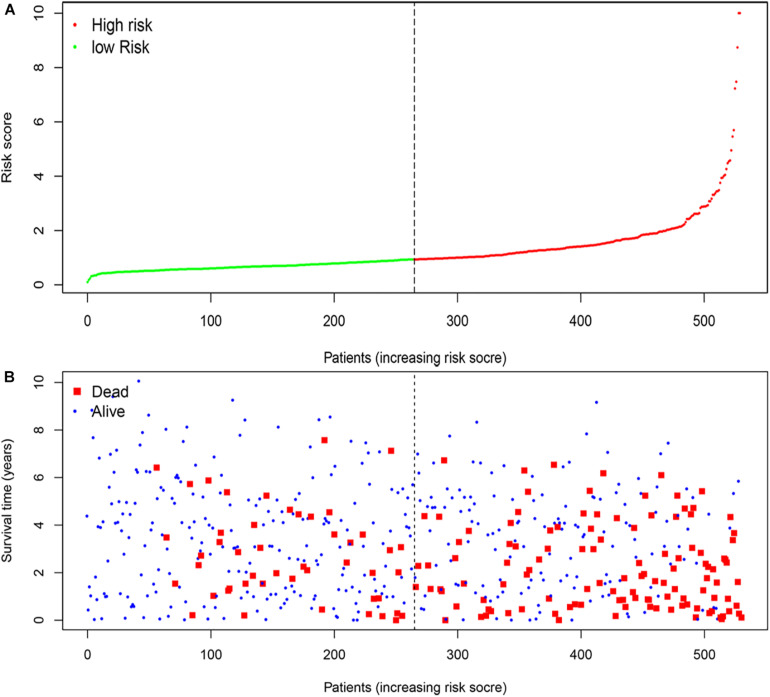
The four-mRNA signatures (B3GAT3, CENPA, AGL, and ALDH3A2) related to risk score predicts the overall survival of patients with ccRCC. **(A)** mRNA risk score distribution. **(B)** Survival time (years) of patients.

According to the median risk score, the patients with ccRCC were classified into high-risk and low-risk groups (Figure 5A). The survival time (years) of each patient was shown in Figure 5B. With the increasing risk score, the number of deaths was also increasing. The risk scores of patients were closely connected with the survival rate.

### Independent Prognostic Analysis of Single and Multiple Factors

**FIGURE 6:**
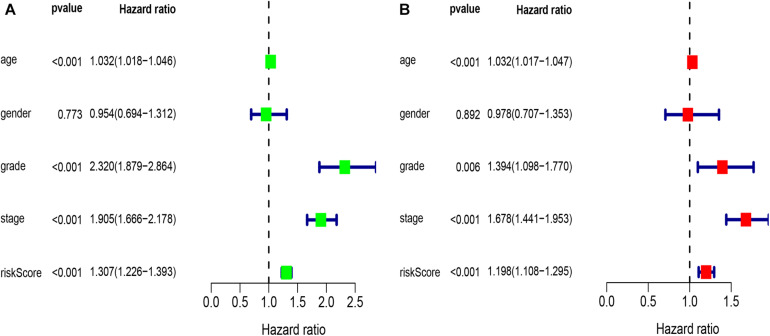
Results of the univariate and multivariate Cox regression analyses regarding OS in the TCGA derivation cohort. **(A)** Results of the univariate Cox regression analyses. **(B)** Results of the multivariate Cox regression analyses.

**FIGURE 7:**
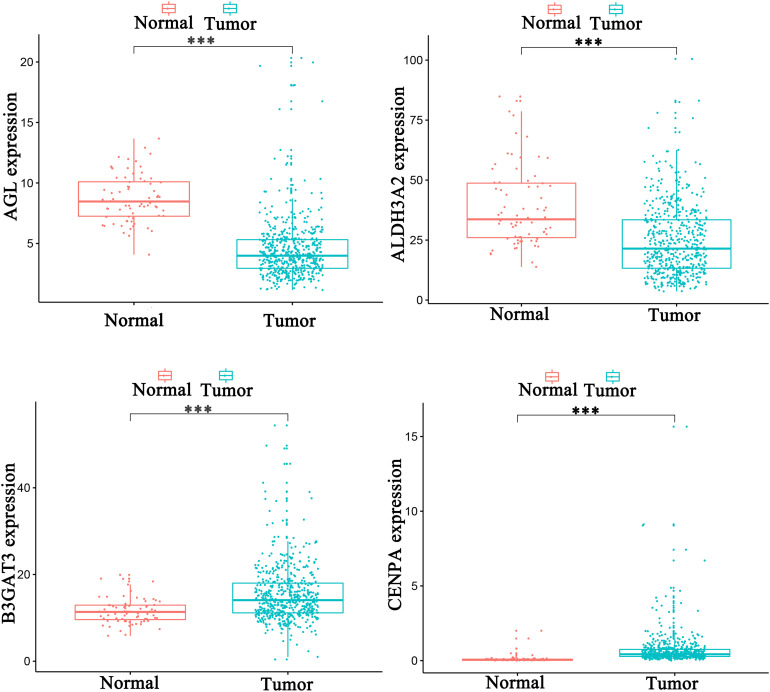
The differential expressions of the four glycolysis-related genes associated with prognostic in paracancerous and ccRCC tissues (^∗^*p* < 0.05; ^∗∗^*p* < 0.01; ^∗∗∗^*p* < 0.001).

**FIGURE 8:**
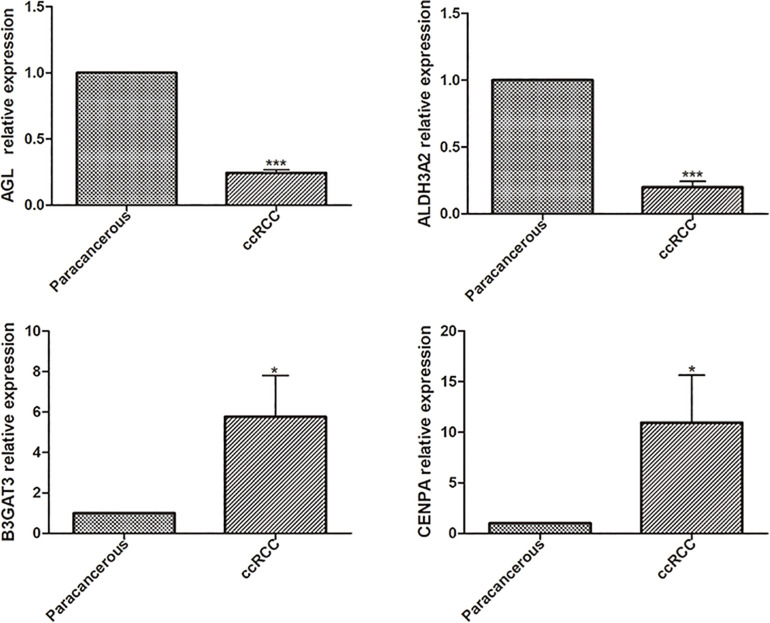
The differential expressions of the four glycolysis-related mRNAs were detected in paracancerous and ccRCC tissues by qPCR (*p < 0.05; **p < 0.01; ***p < 0.001).

Univariate analysis revealed that age, grade, stage, and risk score were adverse prognostic factors for survival (Figure 6A). Multivariable survival analysis also remained that risk score was independent prognostic factors influencing patients with ccRCC (Figure 6B). The differential expressions of the four glycolysis-related genes associated with prognostic in paracancerous and ccRCC tissues were also investigated (Figure 7). The B3GAT3 and CENPA were all significantly upregulated in the tumor tissues. The AGL and ALDH3A2 were all significantly downregulated in the tumor tissues. Meanwhile, the expression of four mRNAs had significant differences in ccRCC and paracancerous tissues by qPCR. The difference of mRNA expression was consistent with the predicted results (Figure 8).

### Validation of Glycolysis Prognosis Model in Predicting Survival of Patients With ccRCC

**FIGURE 9:**
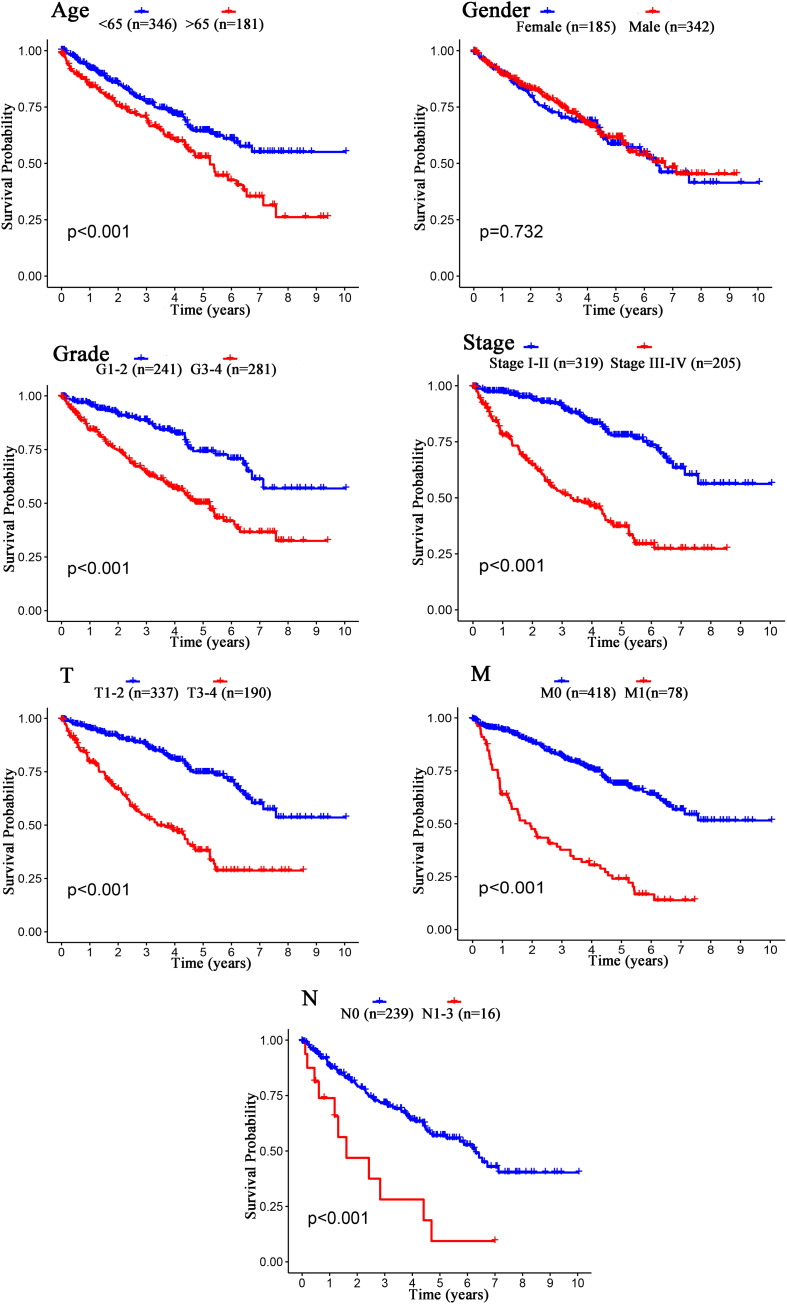
Kaplan–Meier survival analysis for patients with ccRCC grouped by different clinical features (Age, Gender, Grade, Stage, T stage, M stage, and N stage).

**FIGURE 10:**
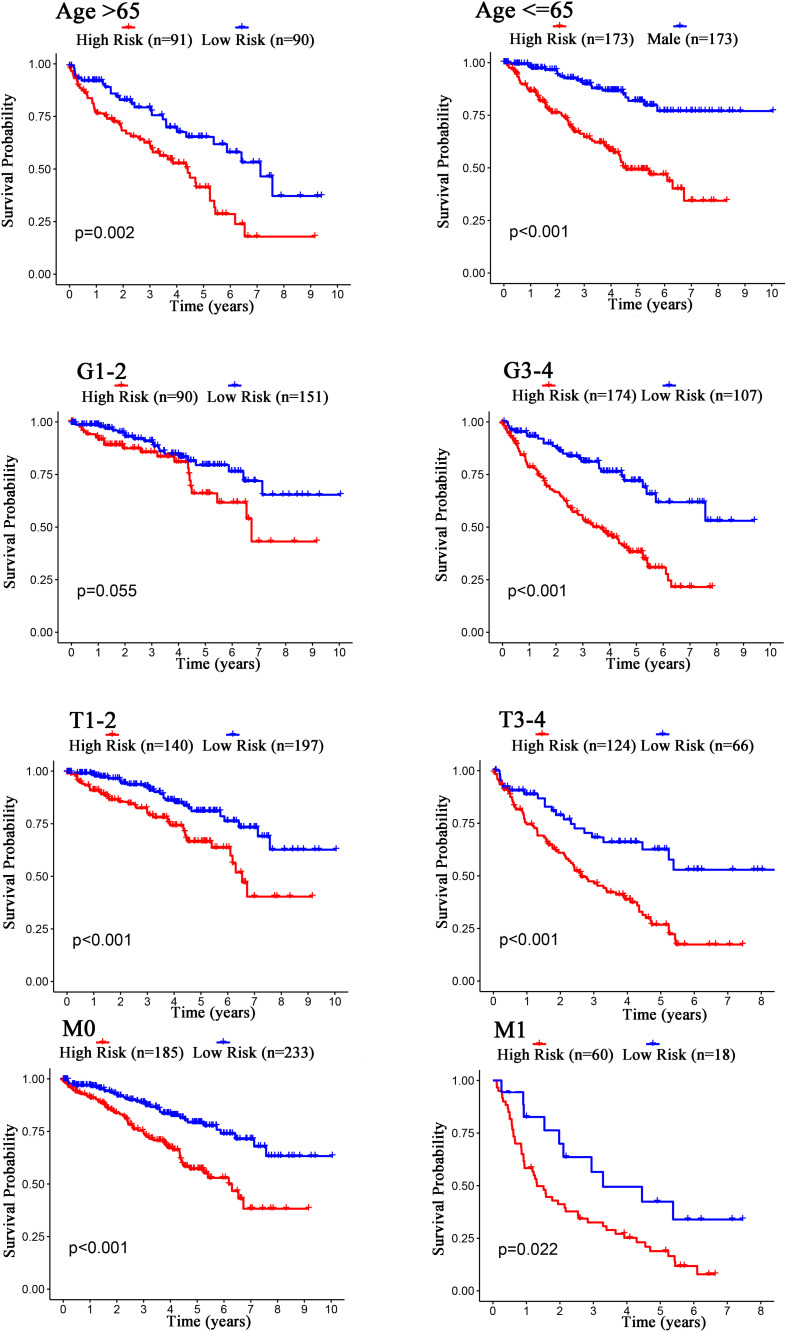
Kaplan–Meier curves for the prognostic value of risk score signature for the patients grouped according to Age, G stage, T stage, and M stage.

**FIGURE 11:**
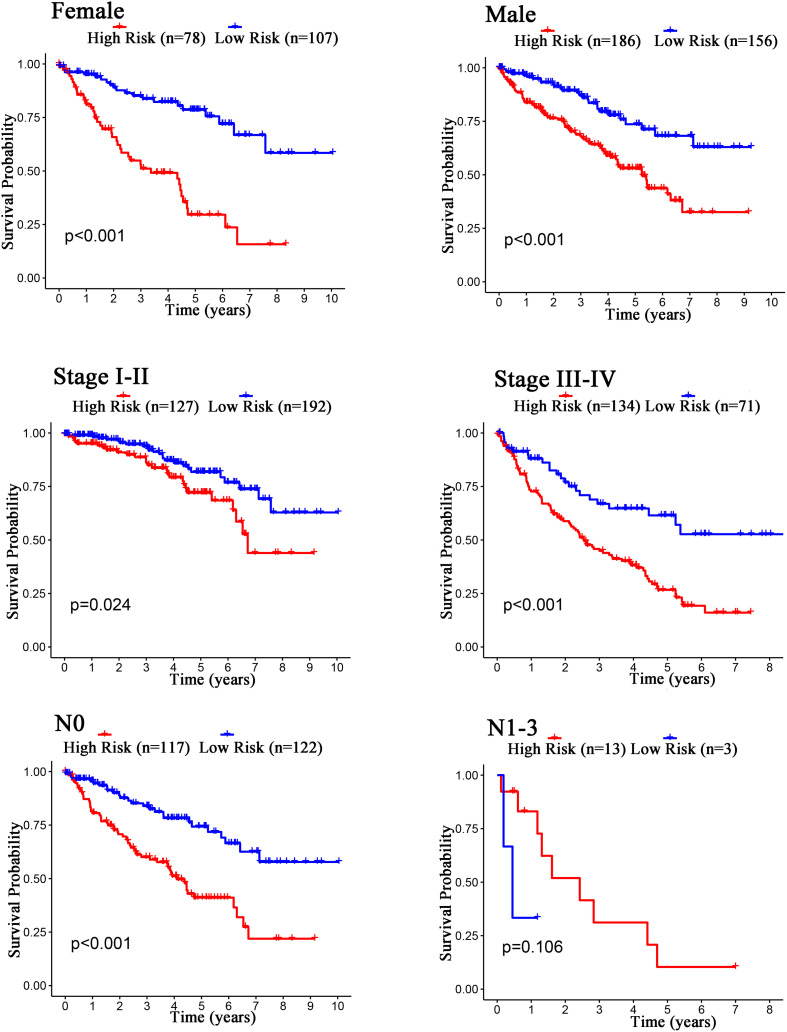
Kaplan–Meier curves for the prognostic value of risk score signature for the patients grouped according to Female, Male, Stage, and N stage.

The Kaplan–Meier curves showed that age, grade, stage, T stage, M stage, and N stage were all significantly correlated with the survival probability of the patients with ccRCC (*p* < 0.001, Figure 9). To verify the risk score was a stable prognostic marker, patients with ccRCC were stratified by age (age > 65 and age ≤ 65), G stage (G1-2 and G3-4), T stage (T1-2 and T3-4), M stage (M0 and M1), N stage (N0 and N1-3), gender (female and male), and stage (stage I–II and III-IV). The patients in the high-risk group had significantly shorter overall survival than those in the low-risk groups in the age > 65, age ≤ 65, G3-4, T1-2, T3-4, M0, M1, Female, Male, stage I–II, stage III–IV, and N0 (Figures 10, 11). However, there was no statistical difference in G1-2 and N1-3 subgroups (*p* = 0.055; *p* = 0.106) (Figures 10, 11). The prognostic power of the risk score in individuals with stage III–IV (*p* < 0.01) was better than that in individuals with stage I–II (*p* = 0.024) (Figures 10, 11).

## Discussion

The recent research progress in energy metabolism has drawn increasing attention from researchers (Dang et al., 2009). There are a lot of undivided relationships between energy metabolism and malignancy (Harami-Papp et al., 2016; Guerra et al., 2017). The normal differentiated cells mainly relied on the oxidative phosphorylation of mitochondria for energy supply, while most tumor cells depend on aerobic glycolysis (Zhu et al., 2020). Unlike most normal cells, tumor cells tended to transform glucose into lactic acid even when oxygen was sufficient to support oxidative phosphorylation of mitochondria (Gatenby and Gillies, 2004; Lunt and Heiden, 2011). The above phenomenon was called the Warburg effect (Warburg, 1956; Heiden et al., 2009; Villar et al., 2015). Although aerobic glycolysis was more detrimental to ATP production than oxidative phosphorylation, aerobic glycolysis increased NADPH synthesis to meet the needs of tumor growth and survival (Otto, 2016). Cancer cells with a high proliferative rate required sustained supplies of macromolecules. Aerobic glycolysis was conducive to provide an abundant supply of necessary substrates for macromolecules by engendering a high glucose flux into cells (Shuch et al., 2013). The application of the isotopic tracer displayed enhanced glycolytic intermediate labeling, suppressed pyruvate dehydrogenase flow, and reduced TCA cycle labeling, consistent with the Warburg effect (Courtney et al., 2018). H Nilsson showed us a strikingly reduced mitochondrial respiratory capacity of primary human ccRCC cells, resulting in enhanced sensitivity to glycolytic inhibition by 3-Bromopyruvate (3BrPA) (Nilsson et al., 2015). ccRCC was basically a metabolic disease. The changes of genes involved in the tricarboxylic acid cycle affected the ability of cells to respond to changes in oxygen, iron and nutrients (Linehan et al., 2019). Although there were many studies on cancer and glycolysis, the research on glycolysis-related cancer biomarkers was limited (Akram, 2013; Ganapathy-Kanniappan and Geschwind, 2013; Gill et al., 2016). In our study, we tried to identify glycolysis-related prognostic biomarkers of patients with ccRCC.

The present study acquired the four biomarker mRNAs (B3GAT3, CENPA, AGL, and ALDH3A2). B3GAT3 was prominently upregulated in ccRCC. B3GAT3 as a member of the glucuronyltransferase gene family played a decisive role in the process of glycolysis (Colman et al., 2019). B3GAT3 catalyzed the formation of the glycosaminoglycan-protein linkage in the process of glucuronic acid transfer reaction (Tone et al., 1999). Abnormal expression of B3GAT3 pushed the speed up process of glycolysis, which contributed to the accelerated proliferation of ccRCC cells and worsen the prognosis of ccRCC patients. Therefore, inhibiting the expression of B3GAT3 could block the glycolysis pathway of tumor cells, which could effectively decrease the proliferation of tumor cells and even kill tumor cells.

As a variant of histone H3 in the centromeric region, CENPA was a key epigenetic factor for the assembly of kinetochore and separation of chromosomes (Howman et al., 2000; Valdivia et al., 2009). The abnormal expression and loss of function of CENPA led to the destruction of genome integrity and abnormal cell division, which led to cancer. Altered expression of CENPA was found in multiple human malignancies including HCC, breast cancer, lung cancer, and colorectal cancer (Tomonaga et al., 2003; Li et al., 2007; Rajput et al., 2011; Wu et al., 2012; Sun et al., 2016). In our study, high expression of CENPA was closely related to the survival of patients with ccRCC. The glycogen debrancher enzyme encoded by AGL was involved in glycogen breakdown (Murray et al., 2003). Inactivation of AGL participated in the pathogenesis of glycogen storage disease. However, studies had confirmed that the role of AGL in tumor biology was independent of its enzyme activity, rather than due to changes in glycogen decomposition (Guin et al., 2014). Darby Oldenburg had further identified that the loss of AGL promoted rapid cancer cell proliferation dependent on extracellular glucose (Oldenburg et al., 2016). In the present study, AGL as a prognostic marker was used to construct the models related to the prognosis of glycolysis in human ccRCC. The expression of ALDH3A2 in ccRCC was less than the paracancerous group. The abnormal expression of ALDH3A2 had a significant impact on the prognosis of patients with ccRCC.

Univariate and multivariate Cox regression analyses were carried out to identify the prognostic value of four gene combinations in patients with ccRCC, rather than a single gene. By comparison with known prognostic biomarkers, this constructed prognosis model may have a more specific and powerful prognostic value in classifying the patients with ccRCC into high and low risk groups. Survival cure showed that patients of the high-risk group had a poor survival rate. The results indicated that the detection and calculation of risk scores were of great significance for prognosis, which could enrich the existing methods for predicting the survival and prognosis of patients.

Most studies had discussed the relationship between glycolysis and tumor cell differentiation, invasion, and proliferation (Fu et al., 2015; Revu et al., 2018; Xiao et al., 2018). The present study firstly used the TCGA database to comprehensively detect and analyze glycolysis-related genes that had important connections with the prognosis of patients with ccRCC. However, the risk score model was established based on the TCGA database and should be validated *in vivo* and *in vitro*.

## Data Availability Statement

The original contributions presented in the study are included in the article/supplementary material, further inquiries can be directed to the corresponding author.

## Author Contributions

CW and XZ contributed significantly to the analysis and manuscript preparation. XC contributed to the verification of glycolysis-related genes. JY, AD, and YC helped to perform the analysis. All authors contributed to the article and approved the submitted version.

## Conflict of Interest

The authors declare that the research was conducted in the absence of any commercial or financial relationships that could be construed as a potential conflict of interest.

## Footnotes

1 https://portal.gdc.cancer.gov/

2 http://www.broadinstitute.org/gsea/index.jsp

3 http://software.broadinstitute.org/gsea/msigdb

4 http://gepia.cancer-pku.cn/
